# Supplementary material for: Inferring individual-level processes from population-level patterns in cultural evolution
Source: R Soc Open Sci. 2017 Sep 6;4(9):170949. doi: 10.1098/rsos.170949 (PMC5627126; doi:10.1098/rsos.170949)
Supplement: Supplementary information [file rsos170949supp1.pdf]

Inferring individual-level processes from population-level patterns in cultural evolution (supplementary material)

Anne Kandler[1,⋆], Bryan Wilder[2,⋆], Laura Fortunato[3,⋆]

[1]Department of Human Behavior, Ecology and Culture, Max Planck Institute for Evolutionary Anthropology
[2]School of Engineering, University of Southern California
[3]Institute of Cognitive and Evolutionary Anthropology, University of Oxford
[⋆]Santa Fe Institute

S1 Rayleigh distinguishability criterion

In addition to the distinguishability criterion described in Section 2.2.2 in the main text, we explored an alternative approach, based on the Rayleigh criterion [1]. This was developed as a criterion for the minimum resolvable detail in optics, e.g. in the case of two images produced by light passing through two separate small apertures. Very generally, it states that two such images are resolvable when the centre of the diffraction pattern of one is directly over the first minimum of the diffraction pattern of the other.

Applied to our problem two transmission modes are considered distinguishable if it holds

$$\bar{t} < a_y \quad \text{with} \quad \int_0^{\bar{t}} f_x(t)dt = 0.5 \quad \text{and} \quad \int_0^{a_y} f_y(t)dt = 1 - \alpha/2, \tag{S1}$$

i.e. the median of probability distribution f_x is realised earlier than the $(1 - \alpha/2)$-quantile of distribution f_y (w.l.o.g. we assume a situation as shown in Figure 1 in the main text).

Condition (S1) poses a rather conservative limit for distinguishability. However, the qualitative outcomes of our analyses did not depend on the criterion used.

S2 ROC curve analysis

Alternative to calculating the conditional probability as shown in equation (2) in the main text, a receiver operating characteristic (ROC) curve analysis could be applied. Given the distribution functions f_x and f_y of the statistic for the transmission modes x and y, the ROC curve is defined by the ratio

$$ROC_{xy}(u) = \frac{\int_u^\infty f_x(v)dv}{\int_u^\infty f_y(v)dv}.$$

Thereby $\int_u^\infty f_x(v)dv$ describes the probability that an empirical estimate larger than u is observed if transmission mode x is acting in the population, while $\int_u^\infty f_y(v)dv$ describes the probability that an empirical estimate larger than u is observed if transmission mode y is acting in the population. In other words, if the value u is used as cut-off point (i.e. for estimate of the statistic has a value larger than u transmission mode x is inferred while for estimates smaller than u mode y is inferred) then $\int_u^\infty f_x(v)dv$ describes the true positive rate and $\int_u^\infty f_y(v)dv$ the false positive rate. Thus the ROC curve is shows the true positive rate as a function of the false positive rates and illustrates the tradeoff between the probability of "detection" and the probability of "false alarm".

The actual shape of the curve is determined by how much overlap O_{xy} the two distributions f_x and f_y have. ROC curves close to the diagonal line point to very similar distribution functions f_x and f_y (corresponding to a large area of overlap) while the closer the curve follows the left-hand border and then the top border of the ROC space, the more different and therefore distinguishable are both distributions (corresponding to a large area of overlap).

Importantly, the gradient

$$\frac{\mathrm{d}ROC_{xy}}{\mathrm{d}u}(u)|_{u=\overline{u}}$$

describes the likelihood ratio between the two hypotheses x and y for a value $\overline{u}$ of the statistic. Additionally, the ROC curve provides a candidate for a rule of thumb for deciding whether transmission modes x and y were acting in the population. The value, denoted by $\tilde{u}$, of the statistic maximizing Youden's index

$$\int_{\tilde{u}}^{\infty} f_x(v)dv + \int_{\tilde{u}}^{\infty} f_y(v)dv = \max_{\forall u}\left(\int_u^{\infty} f_x(v)dv + \int_u^{\infty} f_y(v)dv\right)$$

is an indication of the best possible (but not necessarily good) cut-off point. I.e. values smaller than $\tilde{u}$ would point to transmission mode y while values larger than $\tilde{u}$ to transmission mode x. However, the reliably of this distinction is determined by the size of the area of overlap.

S3 Supplementary results

S3.1 Population-level patterns

In this section we provide examples of the analyses underlying the results presented in Section 3.1 in the main text. The figures show the distribution functions of statistics $p^x_{\max}$, k^x, $d^x_{S'}$, $t^x_{\max}$, conditioned on transmission modes $x \in \{v, h, o, n, m\}$. The joint probability distribution of the frequencies $[p^x_1, \ldots, p^x_5]$ of the five variants of a trait is five-dimensional and cannot be represented in the same way (Section 2.2.1 in the main text).

Each plot corresponds to a given statistic and parameter constellation, showing the distribution function of the statistic generated by 30,000 simulation runs. In each plot, value v of a distribution function at point (u, v) is the probability that the corresponding statistic takes a value smaller than u. For example, if the value v of the distribution function at u is close to 0, then the statistic almost certainly assumes values larger than u. If the value v of the distribution function at u is close to 1, then the statistic almost certainly assumes values smaller than u.

S3.1.1 Pure modes

Figure S1 shows the distribution functions of statistics $p^x_{\max}$, k^x, d^x_S, $t^x_{\max}$, conditioned on transmission modes $x = v, h, o, n$, for parameter constellations $N = 100;\ \mu = 0.1;\ p_{\mathrm{w}} = 0.5, 1$. The left panel in each pair corresponds to $p_{\mathrm{w}} = 0.5$, the right panel to $p_{\mathrm{w}} = 1$.

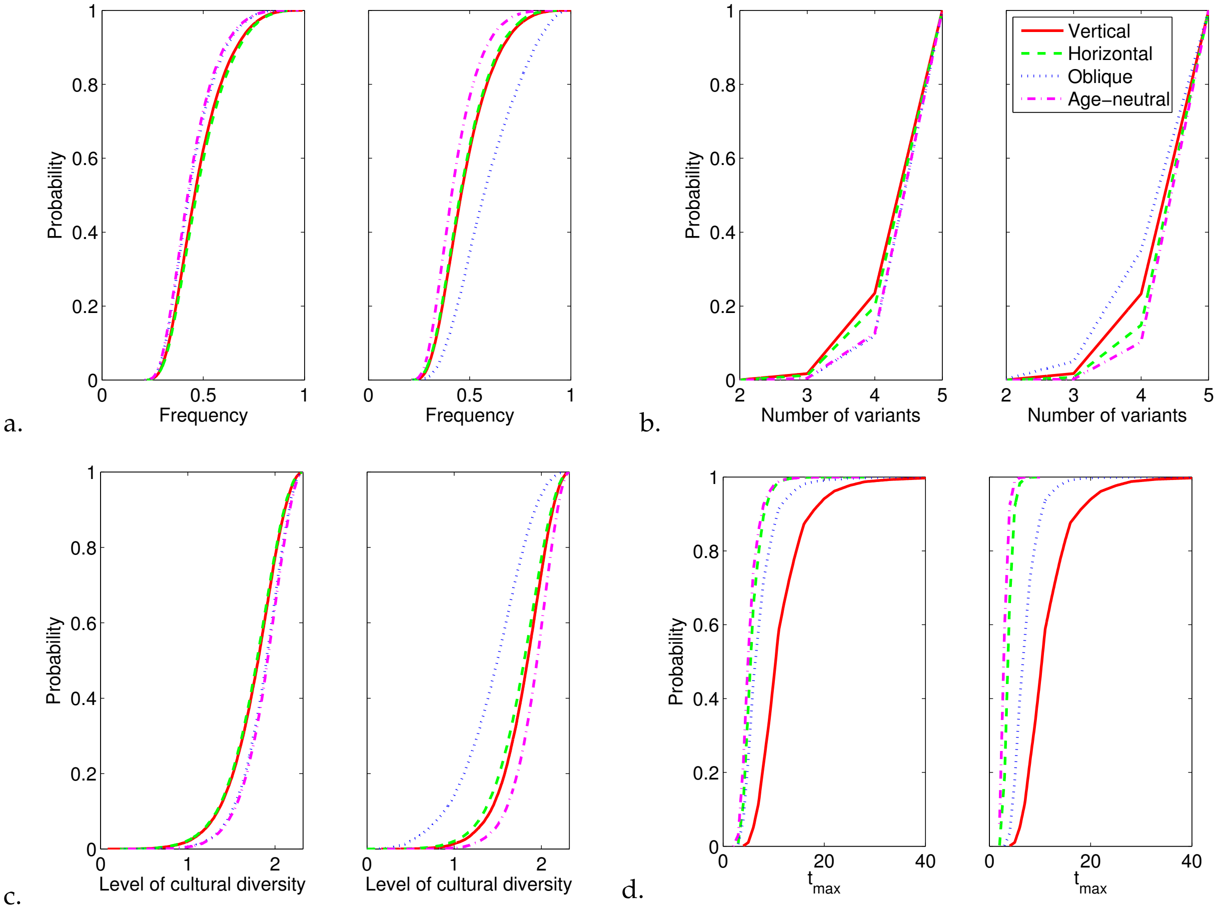

Figure S1: (a) The frequency of the most frequent variant p^x_{max}, (b) the number of variants present in the population k^x, (c) the level of cultural diversity d^x_S, (d) the average time $t^x_{\max}$ a variant stays the most common variant. Shown are the distribution functions for the statistics p^x_{max}, k^x, d^x_S and $t^x_{\max}$ under the parameter constellations $N = 100$, $\mu = 0.1$, $p_{\mathrm{w}} = 0.5$ (left figures of each pair) and $p_{\mathrm{w}} = 1$ (right figures of each pair).

Figures S1(a–c) show the behaviour of statistics $p^x_{\max}$, k^x, d^x_S, which describe the composition of the population at a given point in time (Section 2.2.1 in the main text). For $p_{\mathrm{w}} = 0.5$ (left panels), the distribution functions are relatively close together, indicating that the different transmission modes result in comparable population-level outcomes.

Outcomes for oblique transmission are strongly influenced by the value of p_{w}. For example, Figure S1(c) shows that for $p_{\mathrm{w}} = 0.5$ (left panel), oblique transmission (blue line) results in higher values of d^x_S (i.e. higher levels of cultural diversity) than vertical and horizontal transmission (red and green lines, respectively); the situation is reversed for $p_{\mathrm{w}} = 1$ (right panel). Figures S1(a, b) reveal consistent patterns for statistics $p^x_{\max}$ and k^x. Overall, these findings suggest that when the interaction probability is high, oblique transmission leads to more homogenous cultural compositions than vertical, horizontal, and age-neutral transmission (see Section 3.1 in the main text).

Figure S1(d) shows the behaviour of statistic $t^x_{\max}$, which describes the temporal dynamic of cultural change

(Section 2.2.1 in the main text). For both values of p_{w}, in this case we observe larger differences between the distribution functions. As discussed in Section 3.2 in the main text, this suggests that the temporal dynamic of the transmission modes carries a stronger signature of the underlying transmission process than static characteristics such as the value of cultural diversity at a specific point in time.

S3.1.2 Mixed mode

Figure S2 shows the distribution functions of statistics d_S^x and $t_{\max}^x$, conditioned on transmission modes $x = h, o, m$, for parameter constellation $N = 100$; $\mu = 0.1$; $p_{\mathrm{w}} = 1$; $p_{\mathrm{mix}} = 0.2, 0.5, 0.8$. As discussed in Section 2.1.3 in the main text, the higher the value of p_{mix}, the more likely that transmission is oblique.

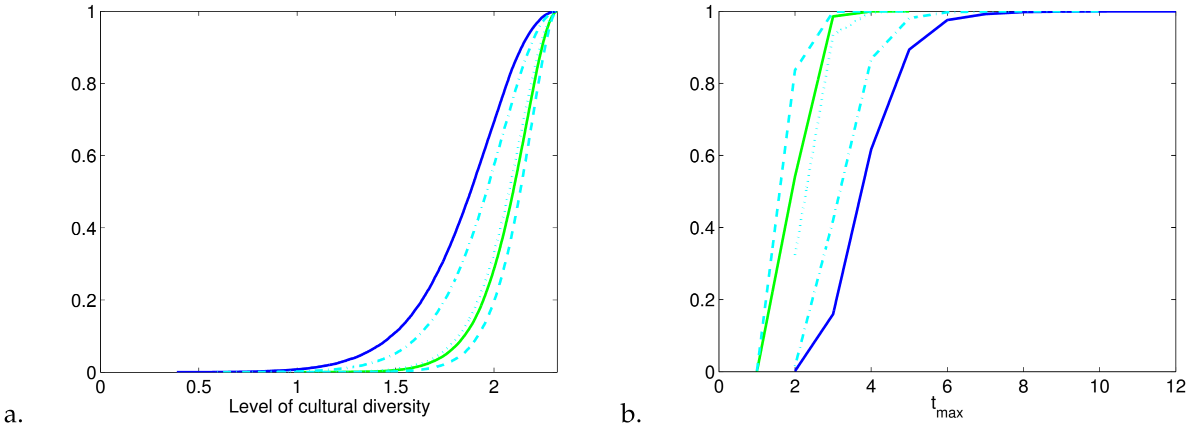

Figure S2: (a) The level of cultural diversity d_S^x and (b) the average time t_{max}^x a variant stays the most common variant in the population. Shown are the distribution functions of the statistics d_S^x and t_{max}^x for all transmission modes under the parameter constellation $N = 100$, $\mu = 0.1$ and $p_{\mathrm{w}} = 1$.

Figure S2(a) shows the behaviour of statistic d_S^x, which summarises the level of cultural diversity in the population (Section 2.2.1 in the main text). For $p_{\mathrm{mix}} = 0.2$, the level of cultural diversity is higher for mixed transmission than for either pure mode (cf. cyan solid line *vs.* green and blue lines). As p_{mix} increases, the level of cultural diversity becomes increasingly comparable to the level for one or the other pure mode — specifically, to horizontal transmission for $p_{\mathrm{mix}} = 0.5$ (cf. cyan dashed line *vs.* green line) and to oblique transmission for $p_{\mathrm{mix}} = 0.8$ (cf. cyan dotted line *vs.* blue line). Overall, at both extremes of p_{mix} the mixed mode results in higher levels of cultural diversity in the population than the "corresponding" pure mode (i.e. horizontal transmission for low p_{mix}, oblique transmission for high p_{mix}).

Figure S2(b) shows the behaviour of statistic $t_{\max}^x$, which describes the temporal dynamic of cultural change (Section 2.2.1 in the main text). Overall, at both extremes of p_{mix} the mixed mode results in a faster rate of change than the "corresponding" pure mode (i.e. horizontal transmission for low p_{mix}, oblique transmission for high p_{mix}).

S3.2 Spreading probability of a mutant

In this section we analyze the spread behaviour of a mutant variant, i.e. a variant with frequency 1, in the population but assume, in contrast to the simulation framework described in the main text, synchronous updating. In particular, we derive the probabilities that the mutant variant possesses frequency m in the next time step. To do so we consider a population of N individuals characterized by the age structure

$$\mathbf{P}(t) = [N_1(t), \ldots, N_5(t)]$$

at time t. The variables $N_i(t)$ denote the number of individuals in age group i. Further,

$$\mathbf{P}^k(t) = [N_1^k(t), \ldots, N_5^k(t)]$$

describes the number of individuals having adopted variant k the five different age groups. It holds

$$\sum_{i=1}^{5} N_i(t) = N(t), \quad \sum_{i=1}^{5} N_i^k(t) = N^k(t) \quad \text{and} \quad \sum_{k=1}^{5} N^k(t) = N(t).$$

The number of transmission events per time step is given by $\overline{N}(t) = \sum_{i=1}^{5} \overline{N}_i(t)$ with $\overline{N}_i(t) = \lceil N_i(t)p_{\mathrm{w}} \rceil$.

For age-neutral transmission we calculate the probability that $m = 0, 1, \ldots, N$ individuals have adopted variant k at time $t+1$ by determining

- the probability that m_1 out of the $N^k(t)$ individual that have adopted variant k at time t are not chosen for cultural transmission (and therefore keep possess variant k in time step $t+1$) and
- the probability that m_2 out of the $\overline{N}(t)$ individuals that have been chosen for cultural transmission have adopted variant k at time $t+1$.

Thereby we consider only combinations of (m_1, m_2) contained in the feasible set

$$V_1 = \{(m_1, m_2) : \max\{0, N^k(t) - \overline{N}(t)\} \le m_1 \le \min\{N^k(t), N(t) - \overline{N}(t)\}, 0 \le m_2 \le \overline{N}(t), m_1 + m_2 = m\}$$

and it holds

$$\begin{aligned} P(N^k(t+1) = m | \mathbf{P}^k(t)) \;\; = & \\ \sum_{(m_1,m_2)\in V_1} & \frac{\binom{N^k(t)}{N^k(t)-m_1}\binom{N(t)-N^k(t)}{\overline{N}(t)-(N^k(t)-m_1)}}{\binom{N(t)}{\overline{N}(t)}} \binom{\overline{N}(t)}{m_2} \left[\frac{N^k(t)}{N(t)}(1-\mu_1) + \frac{N(t)-N^k(t)}{N(t)}\mu_2\right]^{m_2} \\ & \left[1 - \left(\frac{N^k(t)}{N(t)}(1-\mu_1) + \frac{N(t)-N^k(t)}{N(t)}\mu_2\right)\right]^{\overline{N}(t)-m_2} \end{aligned}$$

with the mutation rates μ_1 and $\mu_2 = \mu_1/4$. In more detail, the first term

$$\frac{\binom{N^k(t)}{N^k(t)-m_1}\binom{N(t)-N^k(t)}{\overline{N}(t)-(N^k(t)-m_1)}}{\binom{N(t)}{\overline{N}(t)}}$$

determines the probability that m_1 out of the $N^k(t)$ individuals that have adopted variant k at time t do not undergo cultural transmission. The second term

$$\binom{\overline{N}(t)}{m_2} \left[\frac{N^k(t)}{N(t)}(1-\mu_1) + \frac{N(t)-N^k(t)}{N(t)}\mu_2\right]^{m_2} \left[1 - \left(\frac{N^k(t)}{N(t)}(1-\mu_1) + \frac{N(t)-N^k(t)}{N(t)}\mu_2\right)\right]^{\overline{N}(t)-m_2}$$

determines the probability that m_2 out of the $\overline{N}(t)$ individuals that undergo cultural transmission have chosen variant k during the transmission process. Summing over all feasible combinations of (m_1, m_2) with $m_1 + m_2 = m$ results in the desired probability $P(N^k(t+1) = m | \mathbf{P}^1(t), \ldots, \mathbf{P}^5(t))$.

The probabilities for horizontal and oblique transmission can be derived in a similar manner, however, the age structure of the population has to be considered now. We calculate the probability that $m = 0, 1, \ldots, n$ individuals have adopted variant k at time $t+1$ we determining

- the probabilities that m_{ia} out of the $N_i^k(t)$ individual in age group $i,\ i = 1, \ldots, 5$ that have adopted variant k at time t are not chosen for cultural transmission and
- the probabilities that m_{ib} out of the $\overline{N}_i(t)$ individuals in age group $i,\ i = 1, \ldots, 5$ that have been chosen for cultural transmission have adopted variant k at time $t+1$.

Thereby we only consider combinations $(m_1, \ldots, m_5)$ with $m_i = m_{ia} + m_{ib},\ i = 1, \ldots, 5$ contained in the sets

$$V_2 = \{(m_1, \ldots, m_5) : 0 \leq m_i \leq \overline{N}_i(t),\ i = 1, \ldots 5,\ \sum_{i=1}^{5} m_i = m\}$$

and

$$\begin{aligned} V_{3,i} = \{(m_{i,a}, m_{i,b}) : \ & \max\{0, N_i^k(t) - \overline{N}_i(t)\} \leq m_{i,a} \leq \min\{N_i^k(t), N_i(t) - \overline{N}_i(t)\}, \\ & 0 \leq m_{i,b} \leq \overline{N}_i(t), m_{i,a} + m_{i,b} = m_i\},\ i = 1, \ldots, 5. \end{aligned}$$

For horizontal transmission it holds

$$\begin{aligned} P(N^k(t+1) = m|\mathbf{P}^k(t)) = & \sum_{(m_1,\ldots,m_5)\in V_2} \sum_{i=1}^{5} \sum_{(m_{ia},m_{ib})\in V_{3,i}} \frac{\binom{N_i^k(t)}{N_i^k(t)-m_{ia}} \binom{N_i(t)-N_i^k(t)}{\overline{N}_i(t)-(N_i^k(t)-m_{ia})}}{\binom{N_i(t)}{\overline{N}_i(t)}} \\ & \binom{\overline{N}_i(t)}{m_{ib}} \left[\frac{N_i(t)^k(t)}{N_i(t)}(1-\mu_1) + \frac{N_i(t) - N_i^k(t)}{N_i(t)}\mu_2 \right]^{m_{ib}} \\ & \left[1 - \left(\frac{N_i^k(t)}{N_i(t)}(1-\mu_1) + \frac{N_i(t) - N_i^k(t)}{N_i(t)}\mu_2 \right) \right]^{\overline{N}_i(t)-m_{ib}} \end{aligned}$$

and for oblique transmission

$$\begin{aligned} P(N^k(t+1) = m|\mathbf{P}^k(t)) = & \sum_{(m_1,\ldots,m_5)\in V_2} \sum_{i=1}^{5} \sum_{(m_{ia},m_{ib})\in V_{3,i}} \frac{\binom{N_i^k(t)}{N_i^k(t)-m_{i1}} \binom{N_i(t)-N_i^k(t)}{\overline{N}_i(t)-(N_i^k(t)-m_{ia})}}{\binom{N_i(t)}{\overline{N}_i(t)}} \\ & \binom{\overline{N}_i(t)}{m_{ib}} \left[\frac{\sum_{j=i+1}^{5} N_j^k(t)}{\sum_{j=i+1}^{5} N_j(t)}(1-\mu_1) + \frac{\sum_{j=i+1}^{5} N_j(t) - \sum_{j=i+1}^{5} N_j^k(t)}{\sum_{j=i+1}^{5} N_j(t)}\mu_2 \right]^{m_{ib}} \\ & \left[1 - \left[\frac{\sum_{j=i+1}^{5} N_j^k(t)}{\sum_{j=i+1}^{5} N_j(t))}(1-\mu_1) + \frac{\sum_{j=i+1}^{5} N_j(t) - \sum_{j=i+1}^{5} N_j^k(t)}{\sum_{j=i+1}^{5} N_j(t)}\mu_2 \right] \right]^{\overline{N}_i(t)-m_{ib}} . \end{aligned}$$

To illustrate the differences more clearly we consider a simple example and assume a population with $N = 53$ individuals distributed as [20 15 10 5 3] across the five age groups. Figure S3 shows the probability that a mutant variant has frequency $0, 1, 2, \ldots, N$ in the next time step if the variant is introduced in age group 1 (thick solid lines), 2 (dotted lines), 3 (dashed lines), 4 (dash-dotted lines) and 5 (solid lines) under mutation rate $\mu_1 = 0.05$ and transmission probabilities $p_{\mathrm{w}} = 0.2$ (Figure S3, first row), $p_{\mathrm{w}} = 0.5$ (Figure S3, second row) and $p_{\mathrm{w}} = 1$ (Figure S3, third row). The pink lines show the distributions for age-neutral transmission, the green lines for horizontal transmission and the blue lines for oblique transmission.

We see that for horizontal and especially oblique transmission the probability distribution $P(N^k(t+1) = m|\mathbf{P}^k(t))$ is influenced by the age of the individual introducing the mutation in the population. Unsurprisingly in the oblique case it holds: the older the individual the higher the probability that the mutant variant is still present in the population in the next time step.

Further, for age-neutral transmission the probability that the frequency of the mutant variant increases in the population (i.e. $m > 1$) increases with the transmission frequency p_{w}. However, this is not the case for horizontal and oblique transmission.

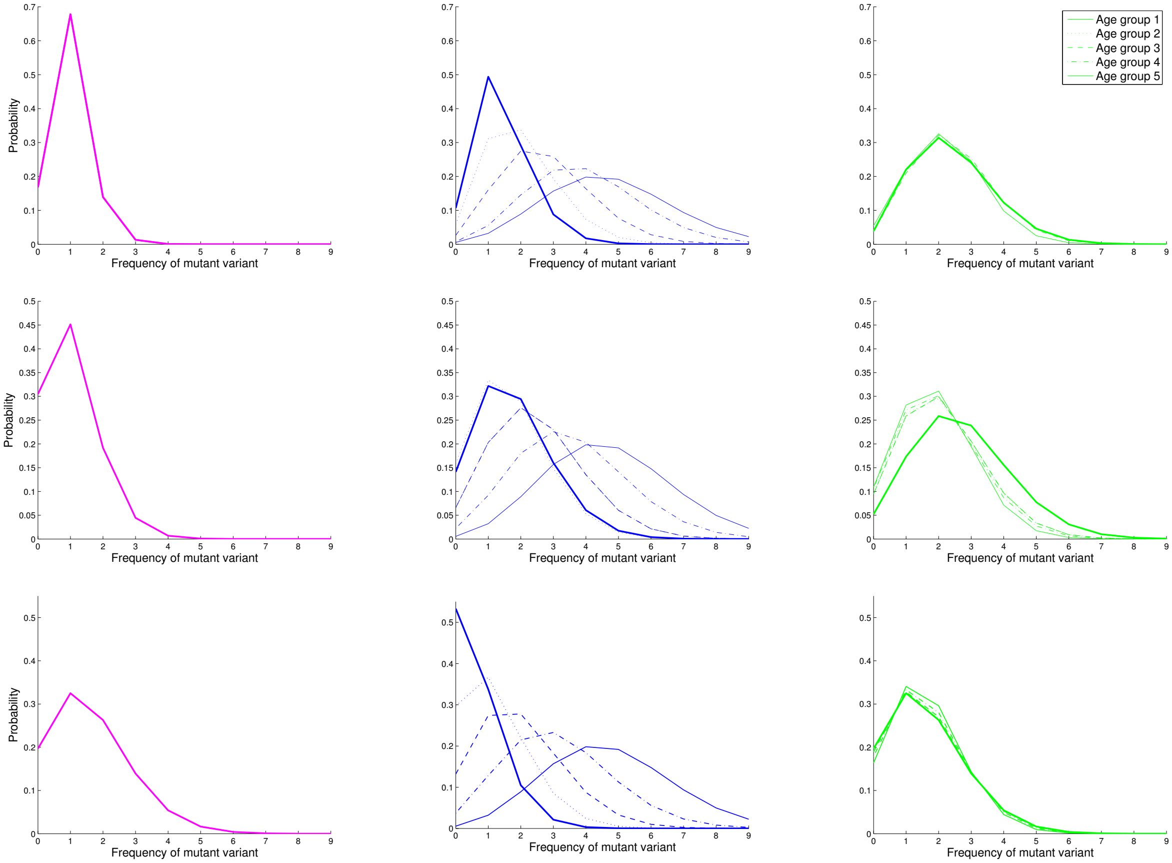

Figure S3: Probability distributions $P(N^k(t+1) = m|\mathbf{P}^k(t))$ that a mutant variant possesses frequency m in the next time step when introduced in age group 1 (thick solid lines), 2 (dotted lines), 3 (dashed lines), 4 (dash-dotted lines) and 5 (solid lines) under the parameter constellations $N = 53$, $\mu_1 = 0.05$, $p_{\mathrm{w}} = 0.2$ (first row), $p_{\mathrm{w}} = 0.5$ (second row) and $p_{\mathrm{w}} = 1$ (third row). The pink lines show the distributions for age-neutral transmission, the green lines for horizontal transmission and the blue lines for oblique transmission.

S3.3 Distinguishability analyses

In this section we present additional results for the distinguishability analyses discussed in Section 3.2 in the main text. The figures show the areas of overlap O_{xy} between two distributions, based on statistics p^x_{max}, k^x, d^x_S, t_{max} for transmission modes x and y, with $x, y \in \{v, h, o, n, m\}$.

The greater the value of O_{xy}, the greater the degree of overlap between the distributions, the less distinguishable they are (Section 2.2.2 in the main text).

S3.3.1 Pure modes

Figure S4 shows the areas of overlap O_{xy} based on statistics p^x_{max}, k^x, d^x_S for all transmission modes, and parameter constellations $N = 25, 50, 100$; $\mu = 0.01, 0.05, 0.1$; $p_{\mathrm{w}} = 0.5, 0.75, 1$.

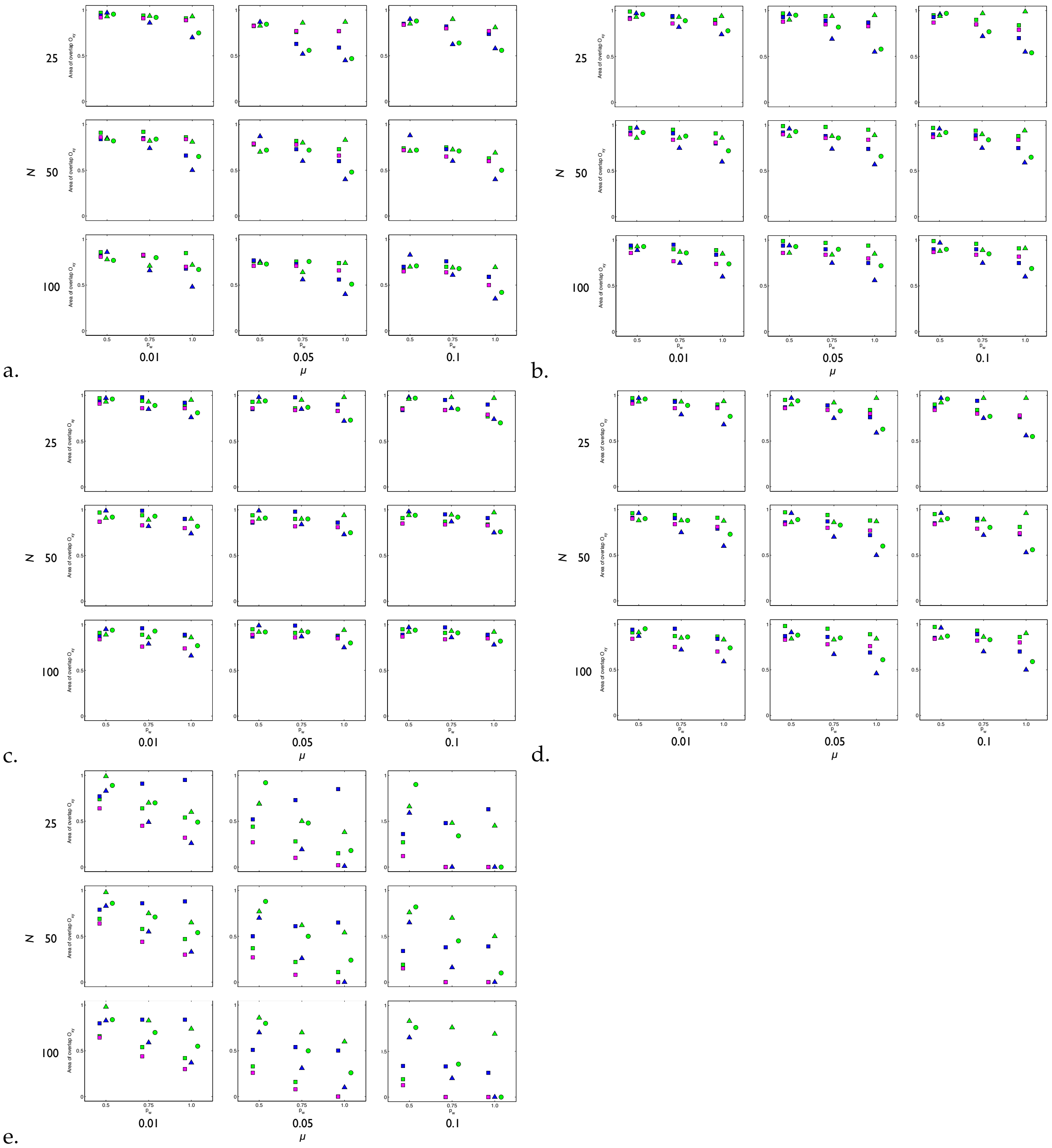

Figure S4: Distinguishability between the pure transmission modes based on (a) the joint probability distribution of the five variants of the trait in the population, (b) the frequency of the most frequent variant p^x_{max} in the population, (c) the number of variants present in the population k^x, (d) the level of cultural diversity d^x_S and (e) the average time $t^x_{\max}$ a variant stays the most common variant. Shown are the values of the area of overlap O_{xy} between the probability distributions of the statistic for pairs of modes, under parameter constellations $N = 25, 50, 100$; $\mu = 0.01, 0.05, 0.1$; $p_{\text{w}} = 0.5, 0.75, 1$. Squares: vertical transmission *vs.* horizontal (green), oblique (blue), age-neutral (pink). Triangles: age-neutral transmission *vs.* horizontal (green), oblique (blue). Circles: horizontal transmission *vs.* oblique.

We have seen in the main text that two pairs of modes result in comparable distributions for the average time a variant stays the most common variant $t_{\max}$ (vertical vs. oblique transmission, age- neutral vs. horizontal transmission) with areas of overlap substantially greater then zero. To provide further insights into these situations we calculated the conditional probabilities $P(x|z)$ given in equation (2) for the pairwise comparison between vertical and oblique transmission and age-neutral and horizontal transmission. Figure S5 illustrates the ROC curve analysis. Figure (a) shows the ROC curves and it is obvious that the distributions of $t_{\max}$ are more similar for horizontal and age-neutral transmission (green line) than for vertical and oblique transmission (blue line). Figure (b) indicates the likelihood ratios for different values of $t_{\max}$. A ratio of 1 means that both transmission modes could have produced the value with equal probability. In agreement with the results from the conditional analysis we see that some values of $t_{\max}$ do not allow for inferences of the transmission mode, but also that there is a directionality in both cases: small values of t_{max} point to age-neutral transmission whereas larger values point to horizontal transmission and a similar statement is true for vertical *vs.* oblique transmission.

The best cut-off point for distinguishing between vertical and oblique transmission is $t_{\max} = 6.2142$ and for horizontal and age-neutral transmission $t_{\max} = 2.7651$.

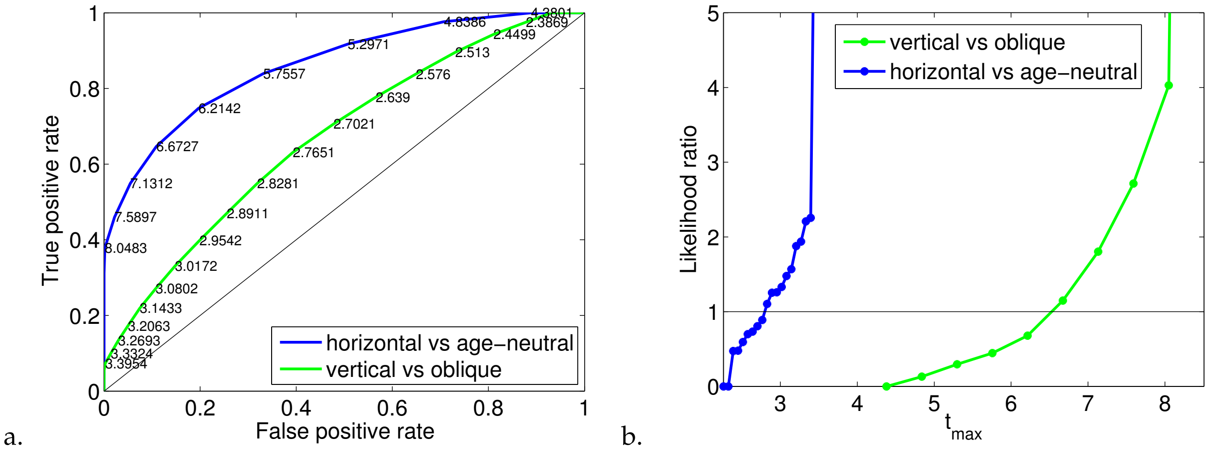

Figure S5: (a) ROC curve for the pairwise comparisons between vertical and oblique transmission (blue curves) and horizontal and age-neutral transmission (green curves), (b) corresponding likelihood ratios based on the average time a variant stays the most common variant $t_{\max}$ under parameter constellations $N = 50; \mu = 0.1;\ p_{\mathrm{w}} = 1$.

S3.3.2 Mixed mode

Tables S1 and S2 quantifies the areas of overlap O_{xy} based on statistics d^x_S and t^x_{max} for all transmission modes $x, y \in \{v, h, o, n, m\}$ and parameter constellations $N = 100;\ \mu = 0.1;\ p_{\mathrm{w}} = 0.5, 1;\ p_{\mathrm{mix}} = 0.2, 0.5, 0.8$.

In this case, we compare distributions of a statistic for (i) the mixed mode at a given level of p_{mix} to the pure modes, and (ii) the mixed mode at a given level of p_{mix} to the mixed mode at other levels of p_{mix}.

Only a limited number of pairs of modes result in distributions that can be distinguished based on the arbitrary threshold $\bar{O} = 0.2$, and only for statistic t^x_{max} (Figure S2). For instance, mixed transmission with $p_{\mathrm{mix}} = 0.5$ can only be reliably distinguished from vertical transmission. Further, at both extremes of p_{mix} the mixed mode cannot be distinguished from the "corresponding" pure mode (i.e. horizontal transmission for low values of p_{mix}; oblique transmission for high values of p_{mix}).

	Mixed p_{w} =0.2	Mixed p_{w} =0.5	Mixed p_{w} =0.8	Vertical	Horizontal	Oblique	Age-neutral
Mixed p_{w} =0.2		0.80	0.82	0.96	0.88	0.97	0.79
Mixed p_{w} =0.5			0.98	0.77	0.83	0.69	0.99
Mixed p_{w} =0.8				0.97	0.79	0.70	0.97
Mixed p_{w} =0.2		0.76	0.62	0.84	0.72	0.87	0.63
Mixed p_{w} =0.5			0.83	0.92	0.93	0.63	0.84
Mixed p_{w} =0.8				0.78	0.91	0.50	0.98

Table S1: Area of overlap O_{xy} based on the level of cultural diversity d_S^x for the parameter constellations $N = 100, \mu = 0.1$, and $p_{\mathrm{w}} = 0.5$ (left tow rows) and $p_{\mathrm{w}} = 1$ (bottom rows).

	Mixed p_{w} =0.2	Mixed p_{w} =0.5	Mixed p_{w} =0.8	Vertical	Horizontal	Oblique	Age-neutral
Mixed p_{w} =0.2		0.6	0.55	0.36	0.69	0.76	0.52
Mixed p_{w} =0.5			0.95	0.14	0.83	0.40	0.78
Mixed p_{w} =0.8				0.11	0.79	0.36	0.95
Mixed p_{w} =0.2		0.48	0.01	0.00	0.01	0.76	0.00
Mixed p_{w} =0.5			0.50	0.00	0.79	0.22	0.48
Mixed p_{w} =0.8				0.00	0.70	0.02	0.98

Table S2: Area of overlap O_{xy} based on the average time t_{max}^x a variant stays the most common variant for the parameter constellations $N = 100, \mu = 0.1$, and $p_{\mathrm{w}} = 0.5$ (tow rows) and $p_{\mathrm{w}} = 1$ (bottom rows).

S3.3.3 Conformity

In figure S6 we compare the probability distributions of statistics d_S^x and t_{max}^x for transmission modes $x, y \in \{v, h, o, n\}$ and parameter constellations $N = 25, 50, 100;\ \mu = 0.01, 0.05, 0.1;\ p_{\mathrm{w}} = 1$. The aim in this case is to compare the distributions of each statistic for a given mode without conformity bias to its conformist counterparts with varying levels of b (i.e. $b = 0$ *vs.* $b = 0.01, 0.02, 0.03$).

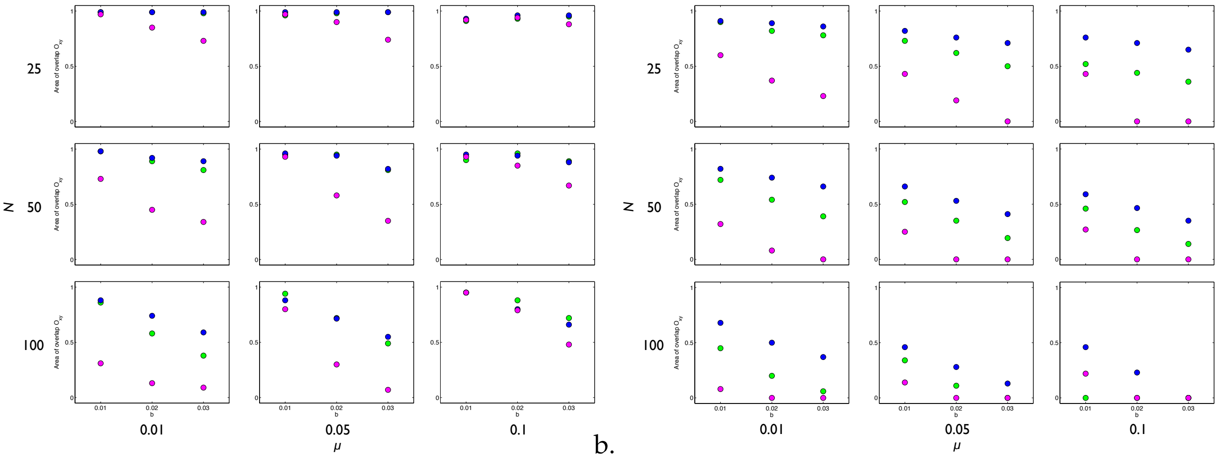

a. b.

Figure S6: Distinguishability between the pure transmission modes and their conformist counterparts based on (a) the level of cultural diversity, d_S^x and (b) the average time a variant stays the most common variant, $t_{\max}^x$. Shown are the values of the area of overlap O_{xy} between the probability distributions of the statistic for a given mode without conformity bias and its conformist counterparts with varying levels of $b = 0.01; 0.02; 0.03$, under parameter constellations $N = 25, 50, 100; \mu = 0.01, 0.05, 0.1;\ p_{\mathrm{w}} = 1$. Green: horizontal transmission. Blue: oblique transmission. Pink: age-neutral transmission.

S3.3.4 Continuous traits

In this section we present summary results of the distinguishability analyses for continuous traits. In our framework, these traits assume any value in the interval $[1, 2]$. We focus on pure transmission modes, denoted V, H, O, N, and equivalent to the corresponding modes in Table 1 in the main text.

In terms of implementation, the key difference between the discrete and continuous cases is in the mutation process (Section 2.1.3 in the main text). For continuous traits, with probability μ a normally-distributed random variable $\omega \sim \mathcal{N}(0, \sigma^2)$ is added to the trait value that an individual inherits from its parent at birth. All traits are initialised at value 1.5.

In the continuous case, the mathematical framework tracks the mean value of a trait across individuals in the population at each time step,

$$m^x = \frac{1}{N}\sum_{i=1}^{N} I_i^x, \quad \text{with } x \in \{V, H, O, N\}.$$

Mean trait values disregard the heterogeneity of the distribution of values across individuals in the population. Consequently, we also investigate discretized versions of the continuous traits. Specifically, the traits are discretized over the interval $[1, 2]$ into two intervals of length $1/2$, three of length $1/3$, five of length $1/5$, and ten of length $1/10$. This produces discrete traits with two, three, five, and ten distinct variants. As in the discrete case, the mathematical framework tracks the frequencies of trait variants at each time step, which can be summarized as described in Section 2.2.1 in the main text.

Analysis of mean trait values In this section we investigate how informative mean trait values are in distinpinkguishing between transmission modes. Figure S7 shows the distribution functions of statistic m^x, conditioned on transmission modes $x \in \{V, H, O, N\}$, for parameter constellations $N = 25, 50, 100$; $\mu = 0.01, 0.05, 0.1$; $p_{\text{w}} = 0.5$. The variance of the mutation process is $\sigma^2 = 0.1^2, 0.05^2$ in Figures S7(a) and (b), respectively.

Overall, the distributions differ in their variances, but they are otherwise highly clustered. This suggests that the transmission modes cannot be distinguished based on the mean trait values. Consequently, mean trait values are not a useful statistic to describe the cultural composition of the population.

Interestingly, for high rates of change and high variance σ^2 of the mutation process (Figure S7b) the distributions of mean trait values are close to uniform over the interval $[1, 2]$. This indicates that the different transmission modes can produce cultural compositions with mean values covering nearly the whole interval $[1, 2]$.

Therefore we conclude that the mean value of a trait does not carry a detectable signature about the underlying transmission modes and might not be an appropriate statistic to characterize the cultural composition of a population.

Analysis of discretized traits Figure S8 illustrates the area of overlap O_{xy} for all combinations of x and y with $x, y \in \{V, H, O, N\}$ based on the average time t^x_{max} a variant stays the most common variant in the population and discretizations in two, three, five and ten variants. While discretizations in two or three variants are not sufficient to ensure distinguishability, discretizations in five and especially ten variants provide more information about the underlying transmission mode. For high rates of change we obtain distinguishability results similar to the discrete case.

This suggests that the discretization of continuous traits should be as fine-grained as possible. Importantly, discretizations in two or three variants (corresponding e.g. to low-high and low-middle-high categorization, respectively) are not enough to ensure reliable distinguishability between different transmission modes. Additionally, the analysis based on the level of cultural diversity shows that we obtain the same result as in the discrete case: vertical, horizontal, oblique and age-neutral transmission are not distinguish-

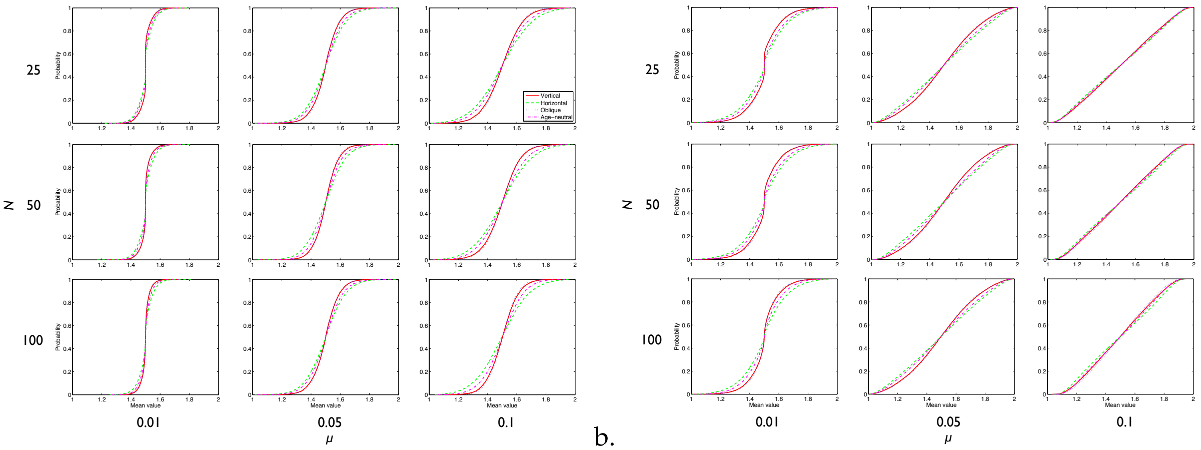

a. b.

Figure S7: The mean value of the cultural trait in the population. Shown are the distribution functions of the statistic m^x for all transmisson modes under the parameter constellations $\mu = 0.01; 0.05; 0.1$, $N = 25; 50; 100$, $p_{\mathrm{w}} = 0.5$ and (a) $\sigma^2 = 0.1^2$ and (b) $\sigma^2 = 0.05^2$ for vertical (red solid lines), horizontal (green dashed lines), oblique (blue dotted lines) and age-neutral (pink dash-dotted lines) transmission.

able on the base of the cultural composition of the populations at a specific point in time according to our distinguishability criterion with $\bar{O} = 0.2$ (see Figure S9).

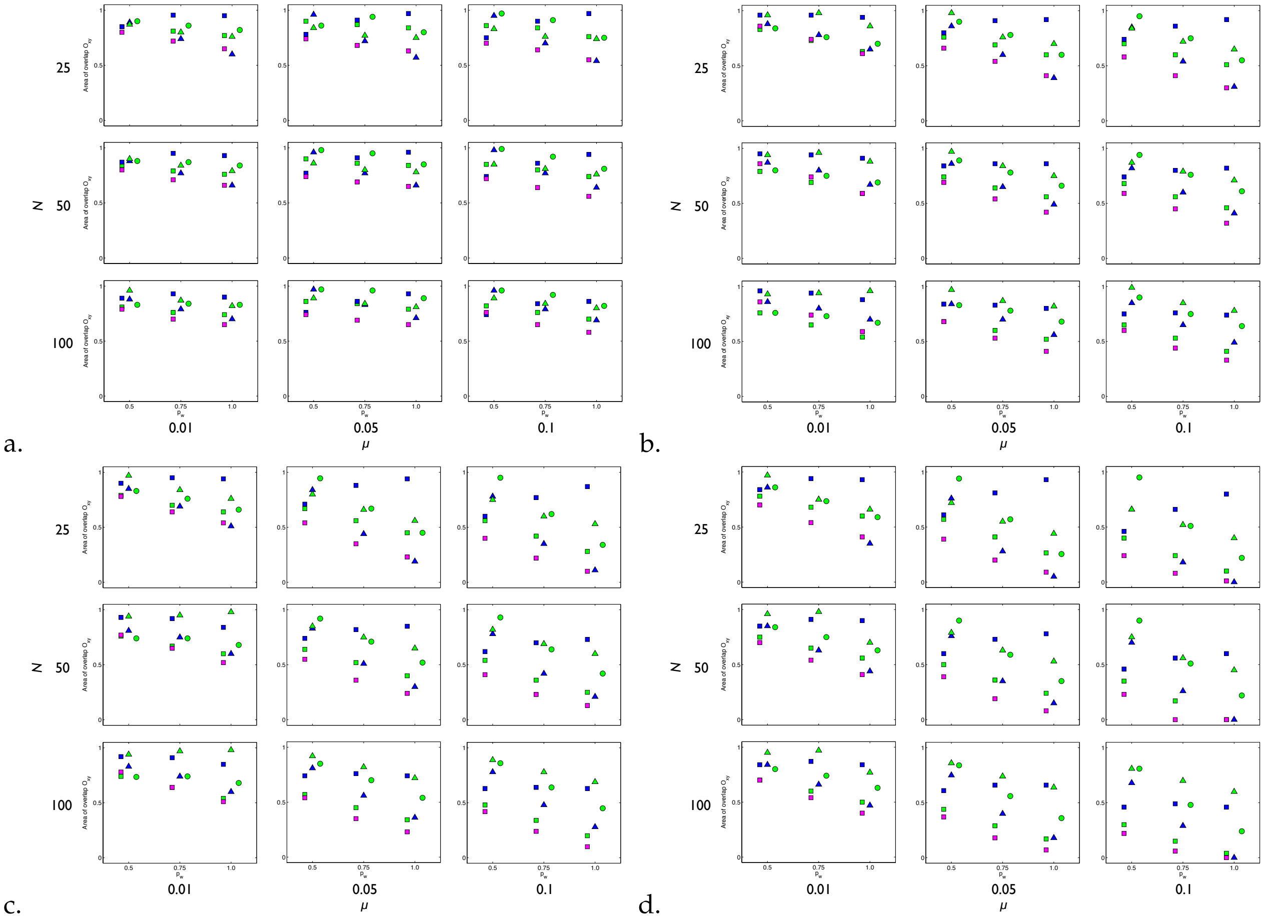

Figure S8: Distinguishability between the pure transmission modes based on the average time t^x_{max} a variant stays the most common variant and discretization into (a) two variants, (b) three variants, (c) five variants, (d) ten variants. Shown are the values of the area of overlap O_{xy} between the probability distributions of the statistic for pairs of modes, under parameter constellations $N = 25, 50, 100; \mu = 0.01, 0.05, 0.1;\ p_{\mathrm{w}} = 0.5, 0.75, 1$. Squares: vertical transmission *vs.* horizontal (green), oblique (blue), age-neutral (pink). Triangles: age-neutral transmission *vs.* horizontal (green), oblique (blue). Circles: horizontal transmission *vs.* oblique.

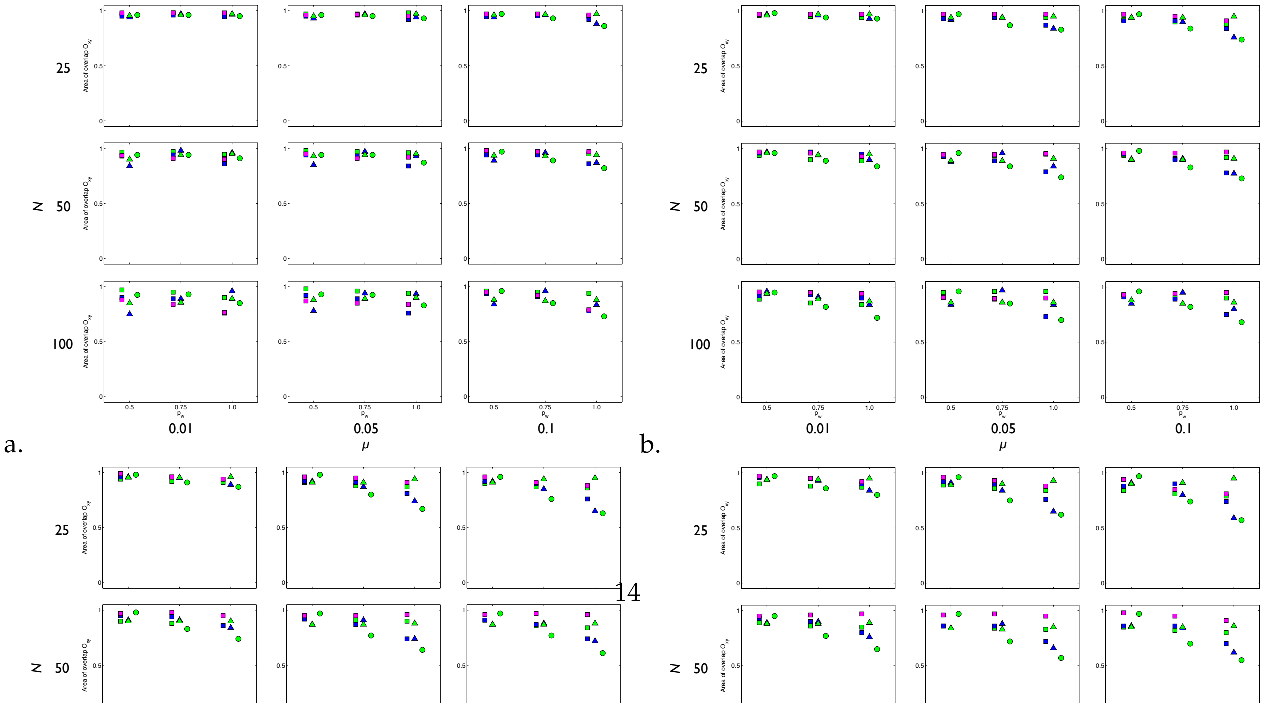

References

[1] Lord Rayleigh. XXXI. investigations in optics, with special reference to the spectroscope. *Philosophical Magazine Series*, 5(8):261–274, 1879.
